# The Galabat-Metema cross-border onchocerciasis focus: The first coordinated interruption of onchocerciasis transmission in Africa

**DOI:** 10.1371/journal.pntd.0007830

**Published:** 2020-02-06

**Authors:** Moses N. Katabarwa, Isam M. A. Zarroug, Nebiyu Negussu, Nabil M. Aziz, Zerihun Tadesse, Wigdan A. Elmubark, Zainab Shumo, Kadu Meribo, Hashim Kamal, Aderajew Mohammed, Yewondwossen Bitew, Tewodros Seid, Firdaweke Bekele, Abebual Yilak, Tekola Endeshaw, Mohammed Hassen, Abate Tillahun, Fikresilasie Samuel, Henok Birhanu, Tadesse Asmare, Daniel Boakye, Sindew M. Feleke, Thomas Unnasch, Rory Post, Tarig Higazi, Emily Griswold, Charles Mackenzie, Frank Richards

**Affiliations:** 1 Health Programs, The Carter Center, Atlanta, Georgia, United States of America; 2 Federal Ministry of Health, Khartoum, Sudan; 3 Federal Ministry of Health, Addis Ababa, Ethiopia; 4 The Carter Center, Khartoum, Sudan; 5 The Carter Center, Addis Ababa, Ethiopia; 6 WHO-ESPEN Lab, Ouagadougou, Burkina Faso; 7 Ethiopia Public Health Institute, Addis Ababa, Ethiopia; 8 College of Public Health, University of South Florida, Tampa, Florida, United States of America; 9 London School of Hygiene & Tropical Medicine, London, United Kingdom, United Kingdom; 10 Department of Biological Sciences, Ohio University, Zanesville, Ohio, United States of America; 11 NTD Support Center, Task Force for Global Health, Atlanta, Georgia, United States of America; University of Liverpool, UNITED KINGDOM

## Abstract

**Background:**

Onchocerciasis transmission across international borders is not uncommon, yet a coordinated cross border stops mass drug administration (MDA) decision has not been documented.

**Methods/Principle findings:**

The Galabat-Metema focus involves neighboring districts on the border between Sudan and Ethiopia. Mass drug administration (MDA) was provided once and subsequently twice per year in this focus, with twice-per-year beginning in Ethiopia’s Metema subfocus in 2016 and in the Sudan’s Galabat subfocus in 2008. Ov16 ELISA-based serosurveys were conducted in 6072 children under 10 years of age in the Metema subfocus in 2014, and 3931 in the Galabat in 2015. Between 2014 and 2016, a total of 27,583 vector *Simulium damnosum* flies from Metema and 9,148 flies from Galabat were tested by pool screen PCR for *Onchocerca volvulus* O-150 DNA. Only 8 children were Ov16 seropositive (all in the Metema subfocus); all were negative by skin snip PCR. The upper limit of the 95% confidence interval (UCL) for Ov16 seropositive was <0.1% for the overall focus and 0.14 positive fly heads per 2000 (UCL = 0.39/2000). However, an entomological ‘hotspot’ was detected on the Wudi Gemzu river in Metema district. The hotspot was confirmed when 4 more positive fly pools were found on repeat testing in 2017 (1.04 L3/2000 flies (UCL = 2.26/2000). Information exchange between the two countries led to stopping MDA in a coordinated fashion in 2018, with the exception of the hotspot at Wudi Gemzu, where MDA with ivermectin was increased to every three months to hasten interruption of transmission.

**Conclusion:**

Coordinated stop MDA decisions were made by Sudan and Ethiopia based on data satisfying the World Health Organization’s criteria for interruption of onchocerciasis transmission. Definitions of entomological ‘hotspots’ and buffer zones around the focus are proposed.

## Author summary

Onchocerciasis is a vector-borne tropical disease caused by the parasitc worm, *Onchocerca volvulus*. The vector is *Simulium* flies that breed in fast flowing rivers and streams. The infection can cause skin disease and loss of vision, giving risk to the common name of ‘river blindness’ for the condition. Ivermectin (Mectizan) mass drug administration (MDA) given at high coverage for many years can interrupt transmission such that MDA can eventually be stopped. The present paper provides highlights of onchocerciasis transmission interruption in the first known coordinated international cross border focus with ivermectin MDA program in Africa, where 99% of the global onchocerciasis burden exists. This focus known as Galabat-Metema is shared between Ethiopia and Sudan, both of which have established a nationwide policy for elimination of onchocerciasis. The paper provides the history of MDA in the border area and the results leading a coordinated end to MDA in the focus in 2017.

## Introduction

Human onchocerciasis is a neglected tropical disease (NTD) caused by a vector-borne parasitic filarial worm, *Onchocerca volvulus* (Nematode: Filaroidea). Adult male and female worms living in subcutaneous nodules produce embryos (microfilariae) that are ingested during a blood meal by day-biting black flies (Diptera: Simuliidae: *Simulium*) that breed in fast flowing rivers and streams. The microfilariae develop in the fly into infective larval stages that can then be transmitted to another person during subsequent blood-feeding. In the human host microfilariae are responsible for a pruritic, often discoloring, dermatitis and visual impairment that may lead to blindness.

In sub-Saharan Africa, mass drug administration (MDA) of the medicine ivermectin (Mectizan, donated by Merck & Co) kills microfilariae, thus reducing skin and eye disease and impeding transmission of the infection. The medicine has little initial impact on the adult worms, and so must be given repetitively over many years. Community-led distribution of ivermectin (referred to as Community Directed Treatment with ivermectin—CDTI) is the main MDA strategy [1]. In the CDTI strategy, community members are educated and empowered to select trusted representatives who are then trained to treat the eligible populations residing in their respective communities. This strategy has successfully been used in Africa, where 99% of onchocerciasis occurs, to deliver ivermectin MDA once or twice-yearly [2, 3].

**Fig 1:**
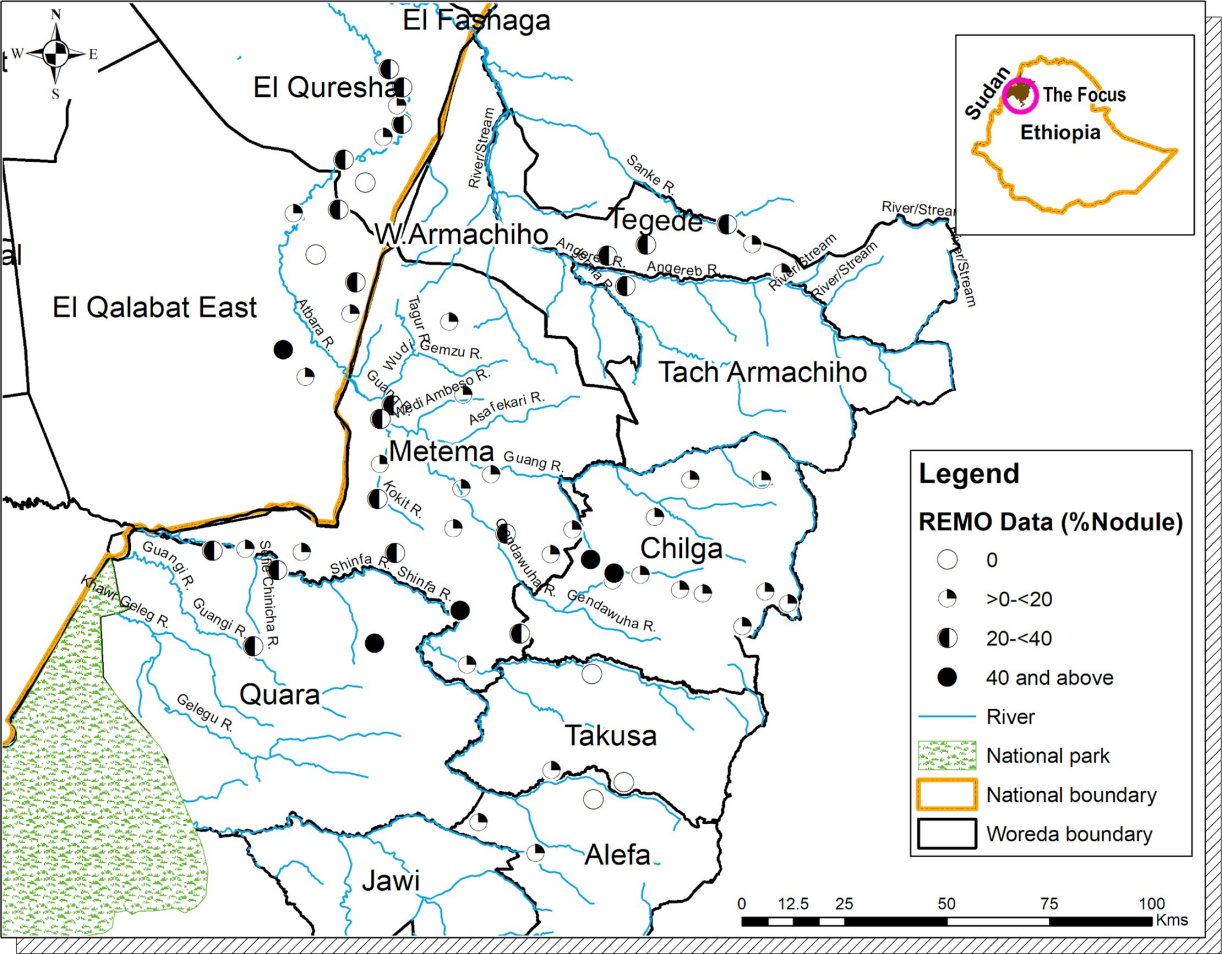
Baseline Rapid Epidemiological Mapping of Onchocerciasis (REMO) conducted in and around the Galabat- Metema Onchocerciasis focus (based of 1998–2011 nodule prevalence surveys, see Table 1. Not copyrighted, and was created in ArcGIS.

**Fig 2:**
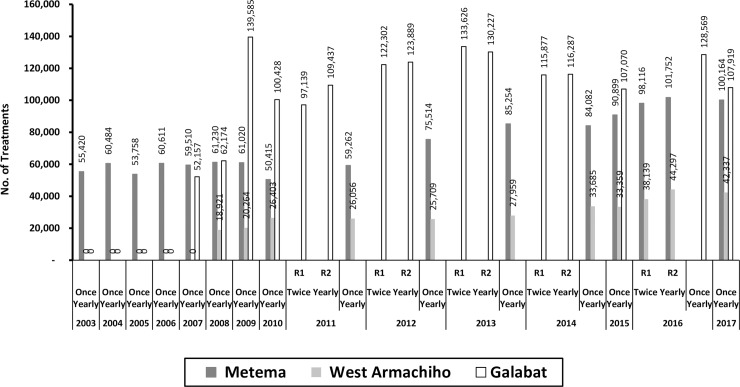
Number of MDA ivermectin treatments provided once or twice yearly in Galabat sub-focus (Sudan) and Metema sub-focus in Metema and West Armachiho districts of Ethiopia) - 2003–2017. Not copyrighted, and was created in Ms Excel.

Onchocerciasis transmission zones may cross international borders and so present a unique challenge of coordination between the different national program activities on each side of the border. We refer to such areas as ‘Special Intervention Zones’ (SIZs) borrowing from terminology used by the now closed Onchocerciasis Control Program of West Africa (OCP). SIZs in the OCP context referred to transmission zones that required unique solutions to address special programmatic challenges (at times including those related to cross-national border issues) [4]. The Galabat-Metema onchocerciasis focus is a transmission area comprising two ‘subfoci’ in neighboring countries: Galabat in Gadarif State of Sudan and Metema in North Gondar Zone of the Amhara Region of Ethiopia-Figs 1 and 2. This SIZ requires special consideration and coordinated operations in the context of the two national elimination programs. Fortunately, Sudan and Ethiopia each has a national policy for nationwide onchocerciasis transmission elimination using twice-per-year ivermectin MDA. Sudan launched its elimination policy in December 2006, while Ethiopia did so in 2012 [3, 5].

The present paper describes a collaborative effort between Sudan and Ethiopia to interrupt transmission of onchocerciasis in the Galabat-Metema focus. We will present the history of the two programs, their ivermectin MDA activities and results from parasitological, serological, and entomological surveys conducted between 2014 and 2017 that resulted in a coordinated 2017 decision to stop MDA, in accord with 2016 World Health Organization (WHO) guidelines [6]. We also briefly describe the Sudanese decision to continue MDA in Galabat until Ethiopia completed its transmission assessments, and Ethiopia’s decision to continue treatment activities in an entomological ‘hot-spot’ in Metema district called Wudi Gemzu. Finally, the lessons learned with respect to buffer zones between these and other onchocerciasis foci, and operations in a Special Intervention Zone will be discussed.

### Onchocerciasis epidemiology and MDA in the Galabat-Metema transmission zone

Onchocerciasis as a cross border phenomenon on the border between Sudan and Ethiopia was recognized by the WHO World Bank African Programme for Onchocerciasis Control (APOC) supported Rapid Epidemiological Mapping of Onchocerciasis (REMO) exercises conducted between 1998–2000 (Table 1 and Fig 1).

**Table 1:** Rapid Epidemiological Mapping for Onchocerciasis (REMO) conducted in Galabat and Metema between 1998 to 2011.

Country	District	Year REMO was Conducted	No. of Villages surveyed	Mean Nodule Prevalence (%)	Range (%)
Sudan	Galabat	2000	5	5.6	0–18
Ethiopia	Metema	1998–2000	17	12	14–44
	Chilga	2000–2001	12	14.6	3–46
	Quara	2000–2001	4	31	27–41
	Tach Armachiho (West Armachiho)	2001	1	14	NA
	Tegede	2011	6	22.8	4–37
	Alefa (Takusa)	2011	3	6	0–14
**TOTAL**			48	15.14	0–46

NA = Not Applicable

REMO data based on nodule rates showed a prevalence range of 14–44% in 17 villages in the Metema district and 0–18% among 14 villages in Galabat. The area was designated the Galabat-Metema focus consisting of two subfoci: 1) the Galabat ‘subfocus’ encompassing the political boundaries of Galabat (now split into Galabat and El Quresha) and 2) the Metema ‘subfocus’ comprising Metema and West Armachiho district. The population in the Galabat subfocus was 146,536 in 2017, while that of the Metema subfocus was 173,923: the entire Galabat-Metema SIZ has a population of 320,459.

**Fig 3:**
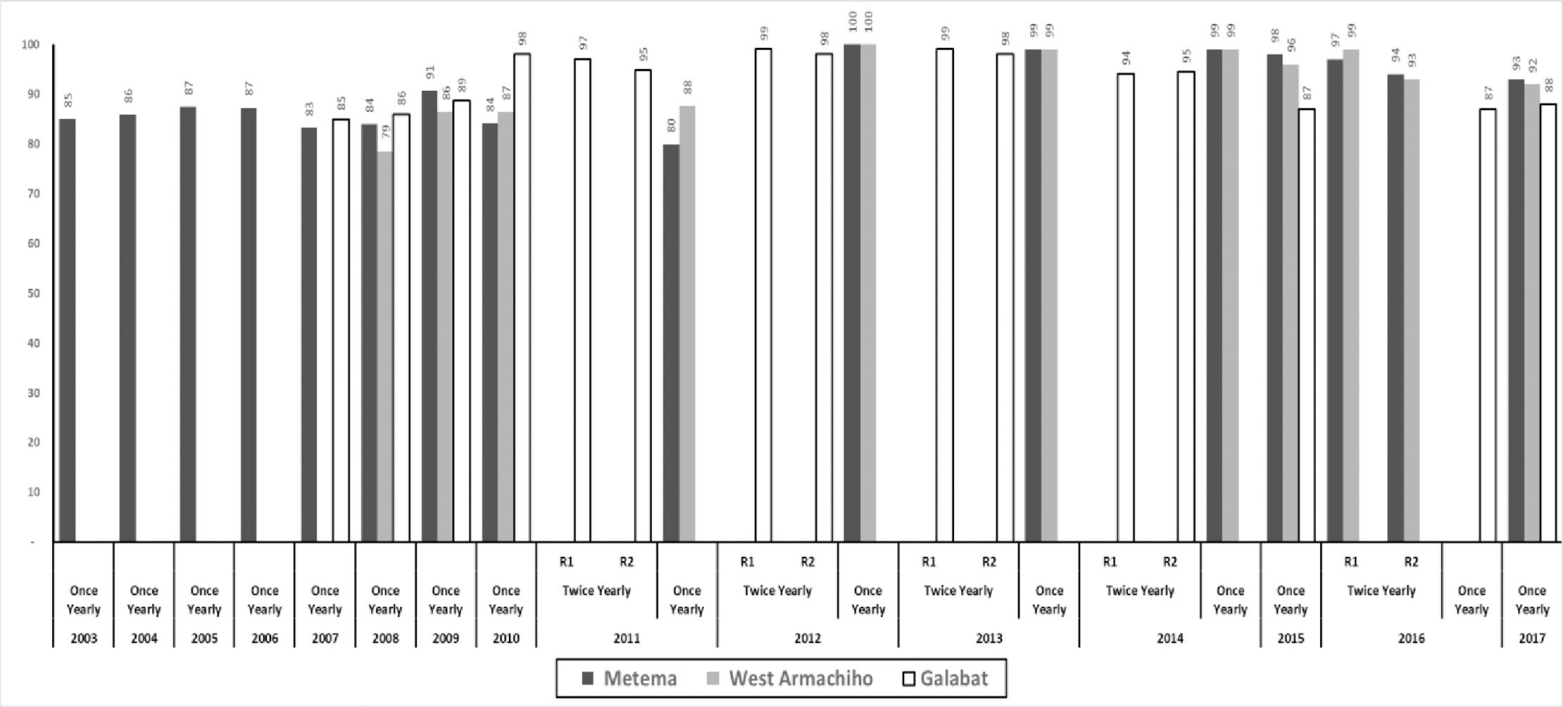
Treatment Coverage of the eligible population of ivermectin MDA provided once or twice yearly in Galabat subfocus and The Metema subfocus (Metema and West Armachiho districts), by year (2003–2017). Not copyrighted, and was created in Ms Excel.

Onchocerciasis in Sudan’s Galabat subfocus was first documented in 1975 [7]. In this area, onchocerciasis presents as a severe form of skin disease characterized by darkened skin (‘sowda’) associated with severe pruritus [8]. Sowda is a localized and usually asymmetrical [9–11]. In sowda, the number of microfialariae (mf) in skin is very low compared to other onchocerciasis regions of sub-Saharan Africa [12]. In the original description, mf prevalence (determined by skin snip examination of 40 individuals in Mushar Ghanam and Sundus communities) was 52.5% [13]. This study was followed by another study in 1985 in which 63% of 173 persons assessed had sowda [8]. In early 1994, communities in Galabat were involved in ivermectin clinical trials, but treatment was discontinued after the study was completed [14]. Annual MDA in a programmatic setting was finally launched late in 2007 (Figs 2 and 3). Based on a successful experience of twice-per-year MDA in the Abu Hamad focus, MDA in Galabat was increased to twice a year in 2011 until 2014 when the program returned to annual treatment as described later in this paper. The first round of MDA in 2007 reached 52,151 people. Subsequent rounds generally increased in scope, peaking at 133,626 in the first of two rounds in 2013 (Figs 2 and 3**)**; an average of 77,166 people were treated per round. Annual coverage was always below 90% of the treatment goal (defined as 84% of the total population), but twice-per-year coverage exceeded 90% in each round. Treatments in the Galabat subfocus ended in 2017.

Onchocerciasis in Ethiopia’s Metema district was first reported around the same time as was Galabat, in the early 1970s [15]. Studies on sowda in Metema were never conducted, but unpublished reports indicated that onchoceral skin disease was a major problem in the district. Annual MDA was launched in Metema district in 2003 and continued until 2016 when twice-per-year treatment was launched (Figs 2 and 3). MDA continued in the new West Armachiho district that was carved out of Metema district in 2008. In the Metema district the number of treatments ranged from 77,629 persons in 2003 to 142,501 in 2017. Coverage of the eligible population ranged from 80% to 100% with a mean of about 91%. In West Armachiho District, treatments ranged from 18,921 in 2008 to 42,337 in 2017. Treatment coverage of eligible population ranged from 79% to 100% with a mean of about 93%. The second round of 2017 was restricted to the hotspot at Wudi Gemzu (population = 11,592, or 3.6% of the focus), for reasons discussed later. Treatments ended elsewhere in the focus in 2017 in a coordinated fashion with the Galabat subfocus.

## Materials

Serology and entomology assessments were conducted to determine if each of the subfoci (Galabat and Metema) could meet the requirements of the 2016 WHO guidelines for stopping MDA [16–19]. These are demonstrating an 95% Upper Confidence Limit [UCL]) of < 0.1% OV16 antibody prevalence in children aged less than 10 years of age, and a UCL < 1 third-stage *O*. *volvulus* larva/2000 vector black flies (<0.05%). Sampling in each subfocus was conducted to get a minimum of 3000 dried blood spots (DBS), obtained by standard finger stick technique, from children five to just under 10 years of age, and at least 6000 vectors per focus.

### Serological assessments

In the Galabat subfocus, DBS from children were purposively collected in 2015 from 9 communities: a) three known endemic sentinel villages; b) four with a known history of onchocerciasis, and c) two close to the border with Ethiopia. In addition, 21 of 34 first line villages and 5 of 26 second line villages (determined based on REMO guidelines) were randomly selected. A total of 3,931 DBS were collected from children resident in these thirty-nine villages, representing 65% of the total of sixty villages in Galabat subfocus. Testing of the Sudanese samples by OV16 ELISA took place in the onchocerciasis molecular laboratory of the Ministry of Health in Khartoum.

In the Metema sub-focus, the DBS samples from 5 years to <10 year old children obtained in 2014 in two separate field outings. Mobilization with village leaders occurred shortly before each field visit. After communities were mobilized, parents were requested to bring their children to a community center usually deemed central and convenient to all the families. West Armachiho in particular had 13 sub-villages attached to the only four existing indeginous villages (‘kebeles’). In the first round (March 2014), the target sample size was allocated across all villages in Metema District proportionally to the estimated population. Based on the household registers in every village under ivermectin treatment, children from 5 years to <10 years of age appeared to be about 9% of the population. This category of children were then selected by convenience from communities ‘high’ risk for onchocerciasis due to their proximity to rapidly flowing water, and a known history of *Simulium* fly biting.

The second round of testing (July 2014) occurred in parts of northern Metema and West Armachiho districts, focusing on children resident in villages close to the Agbara River (that forms part of the border with Sudan) as well Angereb River in West Armachiho. Villages were again chosen purposively, and after mobilization parents with their children were invited to convenient central locations in the villages for DBS sampling. The target sample size per village was again determined proportionally. Only resident children were selected for DBS in Metema and West Armachiho districts. A total of 6072 children (4,369 from Metema District and 1,703 from West Armarchiho District) were assessed from 27 villages in Metema District and 4 Villages in West Armachiho District. Testing of the Ethiopian samples by OV16 ELISA took place in the onchocerciasis molecular laboratory of the Ethiopian Public Health Institute in Addis Ababa using the same protocol as used in Khartoum.

In the whole Galabat-Metema focus, a total of 10,003 five to under 10-year-old children were tested for the presence of IgG4 antibodies recognizing the Ov16 antigen [20]. DBS from each child were properly labeled before packing, transportation from the field and storage in desiccated plastic bags in a deep freezer (-20°C). In the laboratory, the serum samples eluted from DBS were exposed to plates coated with purified recombinant Ov16–glutathione S-transferase (GST) antigen. Bound antibodies then were detected by exposure to biotin conjugated goat anti-human IgG4, and streptavidin conjugated with alkaline phosphatase. The plates were developed with paranitrophenol phosphate (PNPP) substrate (Sigma Chemical, St. Louis, MO). Putatively positive samples were retested with plates coated with Ov16–GST and with control GST. Samples that provided positive readings in both Ov16 assays and were negative for GST alone were scored as confirmed positive.

From Ov16 positive children, skin snips were collected and analyzed with standard O-150 polymerase chain reaction (PCR) distinguishing patent infection from exposure [21, 22]. In accordance with WHO guidelines, in circumstances where there are under 10 children found to be OV16 positive, those who are also PCR negative do not need to be considered as positive for infection in prevalence and confidence interval (CI) calculations. Confidence intervals (CIs) in sampled locations with no positives were calculated with the Bayes Critical Point from Poolscreen, for locations with confirmed positives, the conventional 95% CI formula was applied [23].

### Entomological assessments

*Simulium* flies were collected according to standard human landing capture procedures from 07:00–18:00 hours during the transmission season in four fly collection sites of Metema District and three of West Armachiho. In Galabat, *Simulium* flies were collected at three sites along the Atbara River. Also, fly collection was done according to standard human landing capture procedures from 07:00–18:00 hours during the transmission season, ten days a month for nine months from June 2014 to February, 2015. [18, 19] The flies were preserved in 95% alcohol in the field, and then taken to the Ethiopia Public Health Institute in Addis Ababa (Ethiopia) and Ministry of Health Khartoum (Sudan) national molecular laboratories, where they were morphologically identified as *Simulium damnosum* complex, placed into pools, and tested by standard O-150 polymerase chain reaction (PCR) to detect *O*. *volvulus* DNA[23]. Aside from pool size, techniques were the generally performed under the same protocol in the two laboratories. The pools analyzed contained only heads that had been separated from the bodies as previously described [24]. The heads were then analyzed using the standard O-150 PCR assay [25]. Positive pools were confirmed by a second PCR. In Galabat, 9,148 *Simulium* flies (comprising 140 pools) collected during 2014/2015 period from three fly collection points were tested. In the Metema area, 27, 583 *Simulium* flies (162 pools) were collected from October 2014 to the end of December 2016 from seven vector collection sites. They were tested in pools containing a maximum of 200 flies. For the entire focus, 36,731 *S*. *damnosum* flies were tested.

The head pools provided an estimate of the infectivity rate (the prevalence of flies carrying only L3 infective larvae in the head), and the Pool Screen software (Version 2.0; University of Alabama, Birmingham, AL) was used to estimate the proportion of positive head pools in the PCR assay and the associated 95% confidence intervals (95% CIs) [25]. Interruption of transmission based on entomological criterion for stopping interventions was defined as per WHO guidelines as the 95% ULCI of <1 infective fly per 2000 (0.05%) flies tested.

### Mapping exercise in adjacent districts

Prior to the assessments mentioned above in Metema sub focus, the status of onchocerciasis in the neighbouring districts was unknown. It was prudent to rapidly determine the status of transmission in the adjacent districts before the stop MDA decision is made. Standard field operating procedures (SFOPs) for mapping onchocerciasis developed by the Federal Ministry of Health (FMoH) were applied [26, 27]. River prospections were done and potential breeding sites along the rivers identified with associated first line “at risk” villages for onchocerciasis selected for sampling. In each village, 100 children (5 to ≤10 years) were included in order to ascertain any recent exposure to onchocerciasis, and 10 adults ≥ 20 years of age (adults resident in their communities for at least 10 years) were selected for collection of DBS. The samples taken from adults provided an indication of long term exposure to onchocerciasis. In each district, three villages were selected. The districts involved were Alefa, Chilga, Quara, Takusa, and Tach Armachiho. A total of 1500 DBS was collected from children and 150 from adults from a total of 15 villages.

Six *Simulium* flies collection sites were established (Alefa-1 site, Chilga-1, Quarra-2, and Tach Armachiho- 2). Fly collection was also done according to standard human landing capture procedures from 07:00–18:00 hours during the transmission over a period of three months from October to December 2016. Fly collection was done two days a week for two weeks per month for about thirteen months from November 2015 to November 2016. DBS and flies collected were processed in Ethiopia as described above and all OV16 positive children were tested by skin snip PCR.

### Ethics statement

The study was approved by the Federal Ministry of Health, Ethiopia and the Emory Institutional Review Board as a routine program monitoring activity and therefore, classified as a “non-research” activity. Before the surveys were conducted, district leaders and health workers were informed of the objectives and importance of the surveys. These informed leaders and health workers then organized and educated the leaders in the selected communities about the planned surveys and their importance in determining whether onchocerciasis transmission had been interrupted. Then the date and time to meet with community members at their respective community centers for health education about onchocerciasis, why surveys were needed to determine the status of transmission, who should be involved, and why informed consent was necessary. During the community meetings, the responsible community leaders and health workers explained the importance of the study and requested verbal informed consent from each selected community. Then after, selected adult subjects were again requested to provide informed verbal consent before being assessed. Every individual had the right to opt out without fear of repercussions. For children, additional informed consent was obtained from their respective parents who accompanied them to the selected centers within their resident communities during the surveys.

## Results

### Serological results

**Fig 4:**
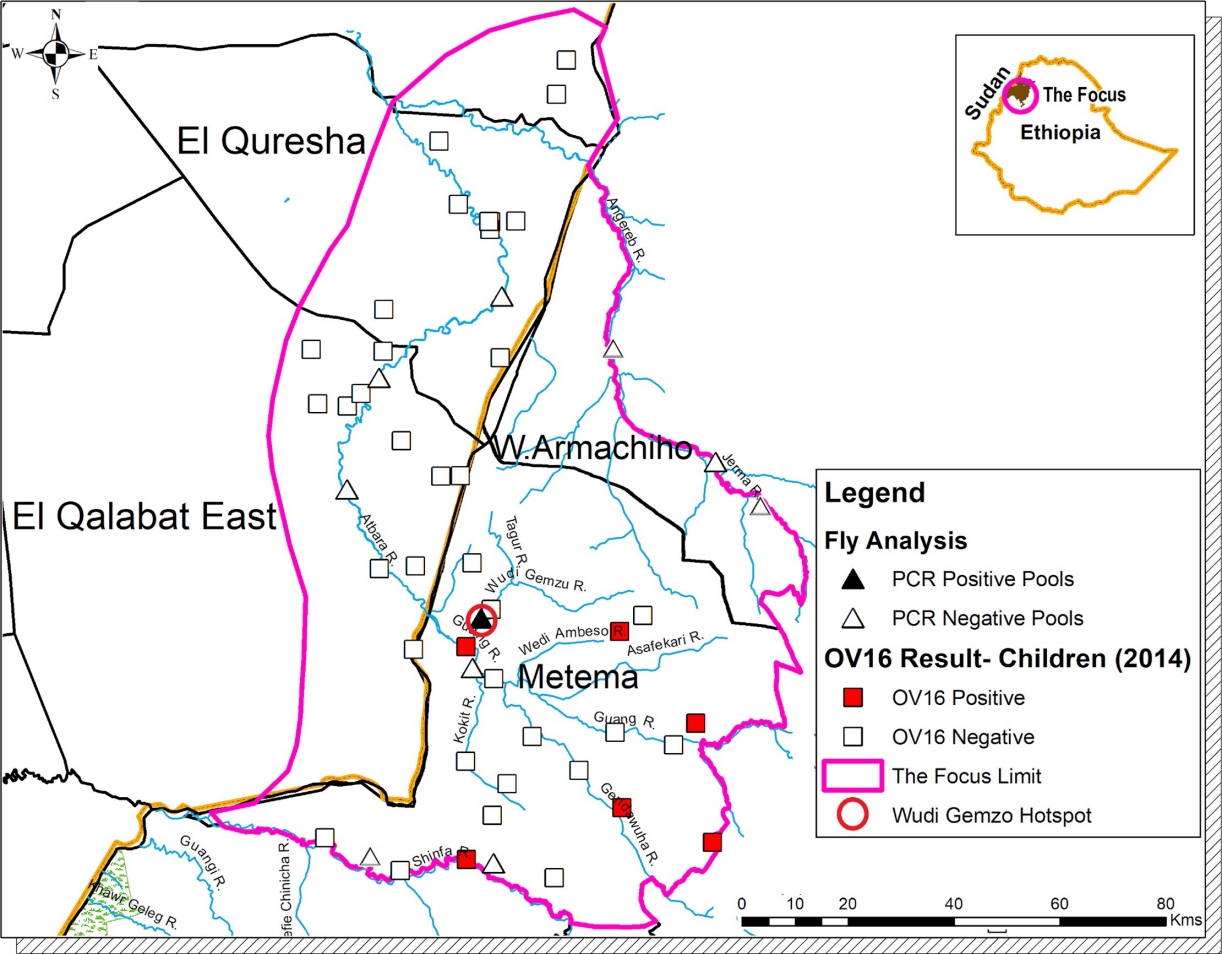
Map of Galabat-Metema focus showing the sites where Stop MDA Ov16 serological and entomological PCR surveys were conducted. ‘Also shown is the Wudi Gemzu Hotspot. The eight OV16 positive children in the transmission zone were all skin snipped PCR negative’. Not copyrighted and was created in ArcGIS.

The evaluation in Metema District in 2014 showed 8 OV16 positive children all of whom tested negative by skin snip PCR (Table 2, and Fig 4). According to 2016 WHO guidelines the OV16 positive children were not to be included in the final tallies. Therefore, results are shown as 0% (95% CI, 0%-0.032%).

In Galabat subfocus, there were also no positive Ov16 ELISA results in the 2015 assessments (95% CI, 0% - 0.049%) (Table 2 and Fig 4). For the entire Galabat-Metema focus the 95% UCL was 0.038%, thus meeting WHO stop MDA serological guidelines.

**Table 2:** Ov16 ELISA and skin snip PCR results from stop MDA assessments in the Galabat-Metema focus (2014–2015).

Country	Locality/ District (year tested)	Ov16 ELISA		Skin Snip PCR on Ov16-positive children		Final Results	
		*Children <10 years old*	*No*. *(%) positive*	*No*. *tested*	*No*. *(%) PCR positive*	*No*. *positive/ No*. *tested*	*Percent positive (95% CI)*
**Sudan**	Galabat (2015)	3931	0 (0%)	-	-	0/3931	0 (0% - 0.049%)
**Ethiopia**	Metema (2014)	4,369	8 (0.26%)	8	0 (0%)	0/4,369	0 (0% - 0.061%)
	West Armachiho (2014)	1,703	0 (0%)	-	-	0/1,703	0 (0% - 0.065%)
	*Ethiopia subtotal*	*6072*	*8 (0*.*13%)*	*8*	*0 (0%)*	*0/6072*	*0 (0% - 0*.*032%)*
**Total**		**10,003**	**8 (0.08%)**	**8**	**0 (0%)**	**0/10,003**	**0 (0% - 0.038%)**

### Entomological results

There were no positive pools for the DNA of *O*. *volvulus* from the 9,148 flies (140 pools) from the Galabat subfocus, (0 L3 per 2000 flies, 95% UCL = 0.42/2000) (Table 3). In the Metema area, although there were 2 of 162 pools (n = 27,583 flies) that were PCR positive, the overall result for the subfocus met the WHO guideline threshold of <1 L3/2000 flies (0.1/2000, 95% UCL = 0.39/2000). Both positive pools were from the collection site on the Wudi Gemzu river **(Table 3 and Fig 4)**. An epidemiological “hot spot” was suspected, prompting collection of an additional 7,884 *Simulium* flies from May to August 2017 at that same site. Analysis of these flies in 81 pools yielded an additional four positive pools (1.04 L3/ 2000 flies, 95% UCL = 2.26/2000). The repeat entomological testing therefore confirmed the 2014 to 2016 period results of a hot spot. Note that the 2017 results from Wude Gemzu are not included in Table 3.

**Table 3:** PCR Results *O*. *volvulus* DNA in *Simulium* flies collected in Gababat (2015) and Metema/West Armachiho (2017).

Country	Locality/District	Lower administrative unit/Kebele	River	Collection point	Latitude (decimal degrees)	Longitude (decimal degrees)	No. of flies analyzed	No. of pools analyzed	No. of positive pools	Point estimate per 2000 flies (95% CI)
**Sudan**	Galabat	Gurasha	Atbara	Maudria	13.56667	36.30000	4,994	76	0	0 (0–0.77)
	Galabat	Atbaraey	Atbara	Hilat Khatir	13.57028	36.01667	2,022	30	0	0 (0–1.88)
	Galabat East	Atbaraey	Atbara	Guaiza	13.41667	36.08333	2,132	34	0	0 (0–1.79)
						*Subtotal*	*9*,*148*	*140*	*0*	*0 (0–0*.*42)*
**Ethiopia**	Metema	Divico	Wude Gemzu	Wude Gemzu	13.00668	36.28284	6,531	40	2	0.41 (0.08–1.13)
	Metema	Gubay	Shinfa	Gubay Jejeb	12.58857	36.24923	1,434	15	0	0 (0–2.62)
	Metema	Shinfa	Shinfa	Shinfa	12.55778	36.09612	1,779	9	0	0 (0–2.34)
	Metema	Meder 6	Guang	Mender 6	12.91806	36.25553	1,000	5	0	0 (0–3.64)
	West Armachiho	Mahrish	Angereb	Mahrish	13.28857	36.66300	7,716	45	0	0 (0–0.49)
	West Armachiho	Abrhajira	Angereb	Abrhajira	13.17665	36.72022	6,259	32	0	0 (0–0.61)
	West Armachiho	Torka	Torka	Torka	13.46517	36.49490	2,864	16	0	0 (0–1.32)
						*Subtotal*	*27*,*583*	*162*	*2*	*0*.*14 (0*.*01–0*.*39)*
**Total**							**36,731**	**302**	**2**	**0.11 (0.008–0.31)**

*Additional testing of vectors from this site were positive by PCR. See text

### Results from the adjacent districts

#### Serology

There were 17 (1.13%) Ov16 positives out of a total of 1,500 children assessed **(Table 4)**. These children were from adjacent districts of Alefa, Chilga, Takusa, and Quara. While the positive children were all skin snip PCR negative, WHO guidelines do not allow more than 10 Ov16 positive/PCR negative children, and therefore we did not proceed to additional sampling for a stop MDA exercise in these districts. There were also 10 (6.67%) Ov16 positive adults from areas south of the Metema subfocus in Alefa (3), Quarra (6) and Takusa (1). Children and adults to the east of Metema (from Chilga and Tach Armachiho districts) were negative.

**Table 4:** 2016 Ov16 ELISA from districts in and around the buffer zone bordering the Metema sub focus.

District	Ov16 ELISA in children <10 years old		Skin Snip PCR on Ov16-positive children		Ov16 ELISA in adults ≥20 years old	
	*No*. *assessed*	*No*. *(%) positive*	*No*. *tested*	*No*. *(%) PCR positive*	*No*. *assessed*	*No*. *(%) positive*
Alefa	300	6 (0.2%)	6	0 (0%)	30	3 (10.0%)
Takusa	300	1 (0.3%)	1	0 (0%)	30	1 (3.3%)
Chilga	300	2 (0.7%)	2	0 (0%)	30	0 (0%)
Quara	300	8 (2.7%)	8	0 (0%)	30	6 (20.0%)
Tach Armachiho	300	0 (0%)	0	0 (0%)	30	0 (0%)
**Total**	**1,500**	**17 (1.13%)**	**17**	**0 (0%)**	**150**	**10 (6.7%)**

**Fig 5:**
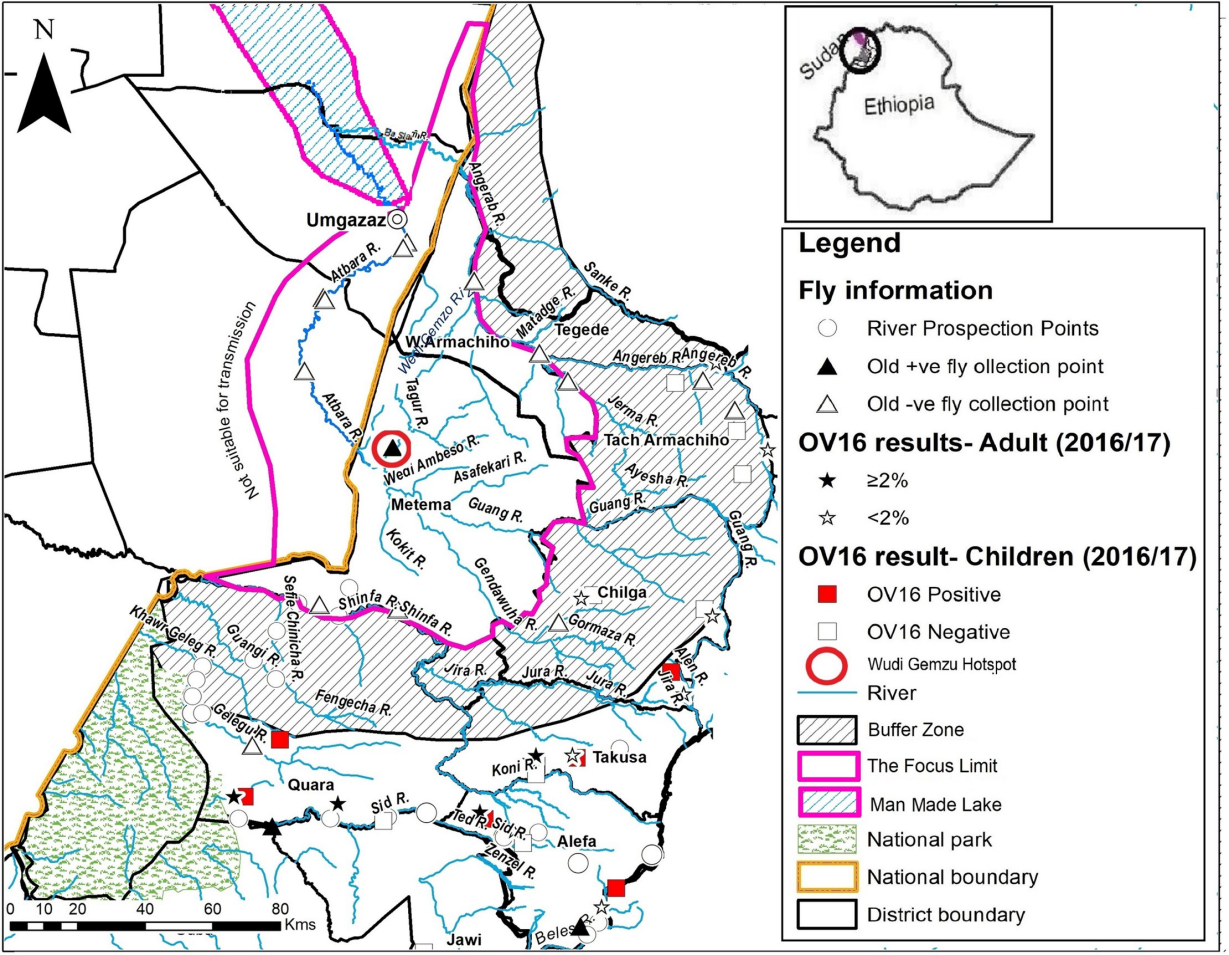
Delineated Map of Galabat- Metema Focus showing the Buffer Zone, the Hotspot, and the Transmission zone where MDA was halted] Not copyrighted, and was created in ArcGIS.

The Ov16 results (shown in Fig 5**)** are not associated with the Metema subfocus, but are found further to the south in an adjacent focus known as the Metekel transmission zone. The area between the Metema and Metekel did not have positive Ov16 samples.

#### Entomology

Overall, 38,160 flies (193 pools) collected from districts adjacent to Metema subfocus yielded 4 positive pools for the DNA of *O*. *volvulus* (0.21 L3/2000 flies, 95% UCL = 0.46/2000), below the WHO threshold. There were two positive pools from southern Quara District and two from Alefa District **(Table 5)**. All positive fly pools were at least 20 km from Metema subfocus limits and within the Metekel transmission zone (**Fig 5**). The area to the south between the Metema subfocus and the Metekel transmission zone, which contained positive fly pools, seemed to be free from onchocerciaisis infection.

**Table 5:** PCR Results for *O*. *volvulus* DNA in *Simulium* flies collected in and around the Buffer Zone (2016 and 2017).

District	Collection point	No. of flies analyzed	No. of pools analyzed	No. of positive pools	Point estimate per 2000 flies (95% CI)
Alefa	Godguadit Silassie	10,862	55	2	0.37 (0.03–1.03)
Quara	Bambaho	26,214	132	2	0.15 (0.011–0.488)
	Gelegu	974	5	0	0 (0–3.75)
Tach Armachiho	Bebew	110	1	0	0 (0–26.6)
	Ashere	0	0	0	-
Chilga	Sinkua	0	0	0	-
	**Total**	**38,160**	**193**	**4**	**0.21 (0.05–0.46)**

## Discussion

### Interruption of transmission in each subfocus, and in the overall cross border transmission zone

Entomological and serological results met WHO criteria for stopping MDA in the cross border Galabat-Metema focus when taken as a whole, or taken individual for subfoci in each country [19]. This decision was taken considering 1) the presence of 8 OV16 positive, but skin snip PCR negative children in Metema, and 2) the presence of an entomological hotspot in Wude Gemzu. Outside the hotspot, the stop MDA decision was made and the rest of the focus was moved in a coordinated and simultaneous fashion to a three-five year Post Treatment Surveillance (PTS) period. A future companion report will describe how the Wude Gemzu hotspot area shown on the maps in this report was delineated using Ov16 and entomological surveys. Based on those surveys the decision was made to continue MDA in a combined population of about 15,000 people with three monthly MDA with ivermectin until transmission interruption is attained.

### Concepts of transmission and buffer zones

According to WHO, a transmission zone is ‘a geographical area where transmission of *Onchocerca volvulus* occurs by locally breeding *Simulium* (black fly) vectors that can be regarded as a natural ecological and epidemiological unit for intervention’[19]. We presumed in this environment that 20 kilometers would be the maximum distance that *S*. *damnosum* s.l. vectors disperse from their breeding sites [28–30]. The area around Metema subfocus limits that was free from onchocerciaisis infection could be referred to as a “buffer” area. The ‘buffer zone’ could be defined as at least a 20 km limit where infected flies cannot cross from one to another transmission zone [28]. Separation of two transmission zones by a buffer zone would theoretically allow for a ‘safe’ stop MDA decision in one transmission zone even if MDA had to continue in a neighboring transmission zone. A buffer zone of at least 20 km around the Metema subfocus has been shown in **Fig 5**.

When the results from entomological and serological surveys conducted in the adjacent districts were plotted, positive results were not found in the buffer zone, but further to the south in the Metekel transmission zone (in southern Quara, southern Takusa and Alefa Districts). The only member of the *S*. *damnosum* complex found in the area was *S*. *damnosum s*.*str*. 'Gondar Form', and likely the vector. The area to the west of Quara District towards the Sudan border, and north of the National Park is uninhabited. The rest of northern Quara and Takusa Districts adjacent to the Metema subfocus have seasonal rivers and the conditions for *Simulium* fly breeding are not favourable. In addition, the absence of infected flies along the southern edge of Metema subfocus was an indication that there are no infected migratory *Simulium* flies from Metekel transmission zone to the south of the buffer zone. Although the REMO map in Fig 1 shows possible onchocerciaisis prevalence in this area, there are a number of factors such as seasonal labour movements during the agricultural season that may have had a drastic impact on transmission in both the buffer zone and possibly neighbouring areas in Metema District. The study recommended more sites for entomological surveillance during the PTS period in order to ensure that no infected flies from Metekel transmission zone invade Metema subfocus or the buffer area unnoticed.

The western and southern part of the Galabat subfocus is surrounded by an arid area with no flowing rivers. To the east and north of the Angereb River in Tsegede District the area in the buffer zone and beyond are uninhabited. For Galabat subfocus in Sudan, the Atbara/Setit dam complex on Atbara River has submerged all the villages up to Khor Hamoda, close to Umgazaz village (Fig 5) [31]. The lake has eliminated all possible fly breeding sites and increased the size of the buffer area on the northern part of the Galabat-Metema focus.

On the eastern side of the Galabat-Metema focus, flies analyzed by PCR were negative for infection, and OV 16 results were negative in children. The demonstration that a “buffer” zone existed around the Galabat-Metema focus increased the confidence of Ethiopia and Sudan ministries of health to stop MDA. The need for close monitoring of the focus during the PTS period was emphasized when this decision was made.

### Coordination of the Galabat and Metema Programs in the cross-border focus

The two programs collaborated by attending each other’s technical meetings, presenting data from each subfocus, and establishing consensus for assessments and recommendations. Periodic joint field visits and synchronized MDA exercises for maximum impact on transmission also were conducted. Each country’s onchocerciasis molecular laboratories were responsible for analyzing their own specimens. However, the laboratories used the same protocol and enjoyed the same technical support and oversight from the University of South Florida WHO onchocerciasis reference diagnostics laboratory. Of particular note is that when Sudan’s assessments in 2015 established that transmission had been interrupted in its Galabat subfocus, the Sudanese program officials agreed to continue annual MDA while waiting for Ethiopia to complete its own stop MDA assessments in Metema subfocus in 2016. This generous offer allowed for a thorough discussion by both parties of the Wude Gemzu hot spot prior to a coordinated stop MDA decision on both sides of the border.

### Special intervention zones (SIZ)

The term “Special intervention zones” was coined just prior to the closure of Onchocerciasis Control Programme (OCP). SIZs referred to zones of ongoing onchocerciasis transmission and morbidity requiring continued programmatic activities [32]. SIZs, many of which were cross border in nature, were funded for continued and heightened interventions under the African programme for Onchocerciasis Control (APOC) through the end of 2007. We propose that the term SIZ be routinely used for cross border transmission areas that are by nature more challenging to eliminate because of the need for binational coordination and special activities [4]. Ethiopia and Sudan (being sovereign nations having different health, financial, security and information sharing policies) presented unique challenges for successful interruption of the cross border Galabat-Metema focus. The close coordination and cooperation of the binational teams were fundamental in forging trusting personal relationships and common interpretation of data needed for Sudan to offer to continue MDA until Ethiopia had completed its assessments, and for both countries to make the joint decision to stop MDA.

## Conclusion

The Galabat-Metema cross-border focus has met the criteria set forth by WHO guidelines for interruption of transmission of O.volvulus. This was accomplished by a combination of annual and semiannual ivermectin MDA. MDA was halted at the end of 2017 in a coordinated binational manner through a process of close collaboration and communication between the governments of Sudan and Ethiopia. Several lessons were learned from this experience: 1) A ‘Special Intervention Zone’ approach is useful to address the unique political dynamics that exist in international border areas. 2) The decision to stop MDA should be supported by peripheral rapid assessments that can demonstrate a ‘buffer zone’ separating the area in question from other areas with ongoing transmission. 3) The Stop MDA decision was made despite the presence of an entomological ‘hotspot’. Intensive (four times per year) MDA is ongoing in this hotspot, as well as comprehensive Post Treatment Surveillance for recrudescence in the remainder of the focus.

## Supporting information

S1 Checklist STROBE statement—Checklist of items that should be included in reports of observational studies. (DOC)
