# Supplementary material for: The Galabat-Metema cross-border onchocerciasis focus: The first coordinated interruption of onchocerciasis transmission in Africa
Source: PLoS Negl Trop Dis. 2020 Feb 6;14(2):e0007830. doi: 10.1371/journal.pntd.0007830 (PMC7004312; doi:10.1371/journal.pntd.0007830)
Supplement: S1 Checklist — (DOC) [file pntd.0007830.s001.doc]

STROBE Statement—checklist of items that should be included in reports of observational studies

|  | Item No | Recommendation |
| --- | --- | --- |
| **Title and abstract** | 1 | (*a*) Indicate the study’s design with a commonly used term in the title or the abstract |
|  |  | The Galabat-Metema cross-border onchocerciasis focus: The first coordinated interruption of onchocerciasis transmission in Africa |
|  |  | (*b*) Provide in the abstract an informative and balanced summary of what was done and what was found  Study considered that status of serology in resident children (under 10 years of age) in order to show the incidence at different points in times during the implementation of mass drug administration with ivermectin. Also, *Simulium* flies were collected for analysis at different times in order to verify the status of infection in the flies. |
| Introduction | | |
| Background/rationale | 2 | Explain the scientific background and rationale for the investigation being reported  The present paper highlights onchocerciasis transmission interruption in the first known coordinated international cross border focus with mass drug administration (MDA) with ivermectin, and verified by the WHO onchocerciaisis elimination guidelines. |
| Objectives | 3 | State specific objectives, including any pre specified hypotheses  The objective was to interrupt transmission of onchocerciaisis using MDA with ivermectin |
| Methods | | |
| Study design | 4 | Present key elements of study design early in the paper  Serological and entomological assessments were conducted to determine whether each of the sub-foci (Galabat and Metema) could meet the requirements of the 2016 WHO guidelines for stopping MDA. These have to demonstrate a 95% Upper Confidence Limit [UCL]) of < 0.1% OV16 antibody prevalence in children aged less than 10 years of age, and a UCL < 1 third-stage *O. volvulus* larva/2000 vector black flies (<0.05%) respectively. Sampling in each sub-focus was conducted to get a minimum sample of 3000 dried blood spots (DBS), obtained by standard finger stick technique, from children five to just under 10 years of age, and at least 6000 vectors per sub-focus. |
| Setting | 5 | Describe the setting, locations, and relevant dates, including periods of recruitment, exposure, follow-up, and data collection  The Galabat area on Sudan side of the border and the adjacent Ethiopia districts of Metema and West Armachiho were study areas. Annual mass treatment in Metema had commenced in early in 2003 was followed by West Armachiho in 2008, and the Ethiopian onchocerciaisis elimination policy was initiated in 2012. In Galabat, MDA commenced in 2007, after the Sudan onchocerciaisis elimination policy had been declared in 2006. Data collection was carried out between 2014 and 2016. |
| Participants | 6 | (*a*) *Cohort study*—Give the eligibility criteria, and the sources and methods of selection of participants. Describe methods of follow-up  *Case-control study*—Give the eligibility criteria, and the sources and methods of case ascertainment and control selection. Give the rationale for the choice of cases and controls  *Cross-sectional study*—Give the eligibility criteria, and the sources and methods of selection of participants; Samples for serology in Galabat area were collected from thirty-nine (65%) villages out of 60 villages. In Metema samples for serology was allocated across all villages in Metema and West Armachiho districts proportional to the estimated population in the entire area under treatment. |
|  |  | (*b*) *Cohort study*—For matched studies, give matching criteria and number of exposed and unexposed: N/A  *Case-control study*—For matched studies, give matching criteria and the number of controls per case: NA |
| Variables | 7 | Clearly define all outcomes, exposures, predictors, potential confounders, and effect modifiers. Give diagnostic criteria, if applicable N/A |
| Data sources/ measurement | 8* | For each variable of interest, give sources of data and details of methods of assessment (measurement). Describe comparability of assessment methods if there is more than one group: N/A |
| Bias | 9 | Describe any efforts to address potential sources of bias: Efforts were made in order to ensure that in Metema/West Armachiho area, every village was represented while in Galabat, 65% of the sixty communities were evenly selected along the Atbara River so that no potential area with likely transmission of onchocerciaisis was left out. |
| Study size | 10 | Explain how the study size was arrived at: In areas selected for sample collection all children under 10 years of age were selected for assessment as per the WHO and national protocols for verification of elimination. Where onchocerciaisis endemicity mapping was done in adjacent districts, 100 resident children and 10 adults were assessed per village as per the Ethiopia national protocol for verification of elimination. |
| Quantitative variables | 11 | Explain how quantitative variables were handled in the analyses. If applicable, describe which groupings were chosen and why: Serology: WHO guidelines were followed. Analysis was done based on 95% CI, and with *Simulium* flies, the point estimate per 2000 flies at 95% CI |
| Statistical methods | 12 | Describe all statistical methods, including those used to control for confounding:  **Serology:** The confidence interval for the population proportion was applied: For sample size is denoted by n, and x denote the number of "positive " in the sample. The proportion for Ov 16 exposure to onchocerciaisis in children was considered as "positive", the outcome of interest. No sign of exposure for Ov16 was "negative." X represented the number of children with an Ov 16 exposure. The sample proportion is p̂ (called "p-hat") is the ratio of positive children to the sample size, that is:   - 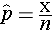   The point estimate for the population proportion is the sample proportion, and the margin of error is the product of the Z value for the desired confidence level (e.g., Z=1.96 for 95% confidence) and the standard error of the point estimate. Therefore, the standard error of the point estimate is:  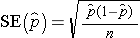  The formula for the point estimate or the best estimate of the proportion of children still with positive Ov16 results. The samples were large and therefore, the confidence interval was computed as below:  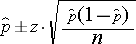  **Entomological PCR Results:** The prevalence of flies carrying infective larvae was computed using the algorithms in the PoolScreen program package (Katholi, C. R., Toe, L., Merriweather, A. & Unnasch, T. R. Determining the prevalence of *Onchocerca volvulus* infection in vector populations by polymerase chain reaction screening of pools of black flies. *J Infect Dis* 172, 1414-1417 (1995)). |
|  |  | (*b*) Describe any methods used to examine subgroups and interactions: N/A |
|  |  | (*c*) Explain how missing data were addressed: N/A |
|  |  | (*d*) *Cohort study*—If applicable, explain how loss to follow-up was addressed  *Case-control study*—If applicable, explain how matching of cases and controls was addressed  *Cross-sectional study*—If applicable, describe analytical methods taking account of sampling strategy: Analysis was done based on 95% CI and *Simulium* flies- point estimate per 2000 flies at 95% CI was considered as per WHO guidelines. |
|  |  | (*e*) Describe any sensitivity analyses |

Continued on next page

| Results | | |
| --- | --- | --- |
| Participants | 13* | (a) Report numbers of individuals at each stage of study—eg numbers potentially eligible, examined for eligibility, confirmed eligible, included in the study, completing follow-up, and analysed |
|  |  | (b) Give reasons for non-participation at each stage: N/A |
|  |  | (c) Consider use of a flow diagram: N/A |
| Descriptive data | 14* | (a) Give characteristics of study participants (eg demographic, clinical, social) and information on exposures and potential confounders: N/A |
|  |  | (b) Indicate number of participants with missing data for each variable of interest: N/A |
|  |  | (c) *Cohort study*—Summarise follow-up time (eg, average and total amount) |
| Outcome data | 15* | *Cohort study*—Report numbers of outcome events or summary measures over time |
|  |  | *Case-control study—*Report numbers in each exposure category, or summary measures of exposure |
|  |  | *Cross-sectional study—*Report numbers of outcome events or summary measures: Out of 10,003 children assessed, only 8 were positive, but later confirmed to be false positive under PCR analysis. For flies 302 pools analysed indicated only two pools in one small area termed as a hotspot was still positive. |
| Main results | 16 | (*a*) Give unadjusted estimates and, if applicable, confounder-adjusted estimates and their precision (eg, 95% confidence interval). Make clear which confounders were adjusted for and why they were included: N/A |
|  |  | (*b*) Report category boundaries when continuous variables were categorized: N/A |
|  |  | (*c*) If relevant, consider translating estimates of relative risk into absolute risk for a meaningful time period: N/A |
| Other analyses | 17 | Report other analyses done—eg analyses of subgroups and interactions, and sensitivity analyses: N/A |
| Discussion | | |
| Key results | 18 | Summarise key results with reference to study objectives: Ov16 ELISA-based serosurveys were conducted in 6072 children under 10 years of age in the Metema subfocus in 2014, and 3931 in the Galabat in 2015. Between 2014 and 2016, a total of 27,583 vector *Simulium damnosum* flies from Metema and 9,148 flies from Galabat were tested by pool screen PCR for *Onchocerca volvulus* O-150 DNA. Only 8 children were Ov16 seropositive (all in the Metema subfocus); all were negative by skin snip PCR. The upper limit of the 95% confidence interval (UCL) for Ov16 seropositive was <0.1% for the overall focus and 0.14 positive fly heads per 2000 (UCL = 0.39/2000). However, an entomological ‘hotspot’ was detected on the Wudi Gemzu river in Metema district. |
| Limitations | 19 | Discuss limitations of the study, taking into account sources of potential bias or imprecision. Discuss both direction and magnitude of any potential bias: The hotspot was confirmed when 4 more positive fly pools were found on repeat testing in 2017 (1.04 L3/2000 flies (UCL =2.26/2000). |
| Interpretation | 20 | Give a cautious overall interpretation of results considering objectives, limitations, multiplicity of analyses, results from similar studies, and other relevant evidence: |
| Generalisability | 21 | Discuss the generalisability (external validity) of the study results:The study results can provide lessons for many onchocerciaisis foci with and without cross border transmission. They are also useful in areas where no vector control has been applied, and hence the importance of a buffer area where knowledge of vector migration into areas where transmission was interrupted is key if disease recrudescence is to be avoided. The decision to stop Mass drug administration in a larger area can be made despite the presence of an entomological ‘hotspot’ as an effective strategy for clearing it has been put in place. |
| Other information | | |
| Funding | 22 | Give the source of funding and the role of the funders for the present study and, if applicable, for the original study on which the present article is based: Although funding for this evaluation was provided by The Carter Center and the Government of Sudan, independent technical committees examined how WHO and national protocols were followed, sample analysis in the lab was done, data was presented. Therefore, the source of funds did not influence the results presented in this paper. |

*Give information separately for cases and controls in case-control studies and, if applicable, for exposed and unexposed groups in cohort and cross-sectional studies.

**Note:** An Explanation and Elaboration article discusses each checklist item and gives methodological background and published examples of transparent reporting. The STROBE checklist is best used in conjunction with this article (freely available on the Web sites of PLoS Medicine at http://www.plosmedicine.org/, Annals of Internal Medicine at http://www.annals.org/, and Epidemiology at http://www.epidem.com/). Information on the STROBE Initiative is available at www.strobe-statement.org.
